# Evaluation of Clinical Outcomes of Negative-Pressure Wound Therapy in Gustilo-Anderson Type IIIA/IIIB Open Fractures of Extremities

**DOI:** 10.7759/cureus.53801

**Published:** 2024-02-07

**Authors:** Arun Kumaar, Arun H Shanthappa, Deepak Hongaiah, Nandini Sanjay, Abhi Sharma

**Affiliations:** 1 Orthopaedics, Sri Devaraj Urs Medical College and Research Institute, Kolar, IND; 2 Plastic Surgery, Sri Devaraj Urs Academy of Higher Education and Research, Kolar, IND; 3 Orthopaedics, Sri Devaraj Urs Academy of Higher Education and Research, Kolar, IND

**Keywords:** open fractures, angiogenesis, wound healing, vacuum-assisted closure, negative-pressure wound therapy

## Abstract

Background

Open fractures are common and serious injuries that primarily affect young males. Fracture management has improved in the last decade. However, infections with their complications are still a concern, especially in open fractures for primary closure of the injured area. A newer technique called vacuum-assisted therapy has become a therapy of choice for many orthopedic surgeons. This study aimed to determine whether vacuum-assisted closure reduces the duration of wound healing and the frequency of infections after fixation of Gustilo-Anderson Type IIIA/IIIB fractures of the extremities.

Methodology

An observational analytical study was conducted among 34 patients with Gustilo-Anderson Type IIIA/IIIB fractures of the limbs who presented to the Department of Orthopaedics, R. L. Jalappa Hospital, Kolar, from December 2019 to July 2021. Negative-pressure wound therapy was employed for wound closure after fixation of fractures. Patients were followed up for one month.

Results

The mean age of the patients was 37.06 ± 10.340 years. The prevalence of infection before vacuum-assisted closure dressing was 80.6%, and the prevalence of infection after vacuum-assisted closure dressing was 19.4%. The difference in proportion before versus after the intervention was statistically significant (p < 0.001) according to the McNemar Test. Hence, vacuum-assisted closure dressing decreased the rate of infection. The mean dimension of the wound before vacuum-assisted closure therapy was 66.05 cm^2^ and the mean dimension of the wound after vacuum-assisted closure therapy was 27.97 cm^2^. The difference in the mean before and after the intervention was statistically significant according to the paired t-test (p < 0.001). Hence, vacuum-assisted closure dressing helped decrease the wound size which was proven statistically.

Conclusions

Vacuum-assisted closure is a viable and beneficial treatment option for complicated fractures with large soft-tissue abnormalities.

## Introduction

Compound fractures are common due to the increasing trend of high-speed motor vehicle accidents [1]. Open or compound fractures are fractures that have an open wound at or closer to the fracture site [2]. Avoidable complications encountered in orthopedic practice are open fractures. In open fractures, the infection percentage of 16-66% has been reported in various investigations [3-10].

As the skin is contravened in open fractures, there is a risk of developing an infection. The Gustilo-Anderson open fracture classification system is commonly used to assess the severity of open fractures [9]. This approach assesses the severity of an injury based on wound size, contamination, and tissue injury. External fixation, debridement, and vacuum-assisted closure (VAC) have been illustrated as management methods [11].

Over the last decade, the practice of negative-pressure wound therapy (NPWT) in treating complex and big wounds has grown in popularity. Modern NPWT systems, which include an open-pore foam sponge, and an adhesive dressing, on top of a vacuum pump that produces negative pressure, have been employed for tissue defects surrounding open fractures and chronic, contaminated wounds as an adjuvant to surgical debridement. Accompanying skin grafts and preventing wounds at the peril of rupturing are two further applications [12].

The use of negative pressure in open wounds is known as VAC, which dramatically promotes wound healing at the macro and micro levels [13]. Vacuum-assisted wound closure can be utilized in place of more traditional wound treatment techniques. The negative pressure allows the wound to heal more quickly or with fewer reconstructive alternatives. VAC has two advantages, namely, it isolates the wound, which reduces the possibility of secondary contamination from the environment, and it minimizes limb edema. The exclusion of edema enhances capillary blood flow, which boosts oxygen and nutrition delivery to the wound [14]. VAC also restricts bacterial growth because of the capillary growth into the wound. This leads to oxygen and immune cells, which fight bacterial growth [15]. VAC has been used to reduce morbidity, cost, and hospitalization duration while also improving patient comfort [16-18].

VAC is a commercially available device that is currently in extensive clinical use as a dressing for various wound types. Although VAC was first employed for open fracture injuries, there is no evidence of its effectiveness among the Indian population [19]. This study aimed to determine whether NPWT reduces the duration of wound healing and decreases the frequency of infections in wound closure after fixation in the treatment of Gustilo-Anderson Type IIIA/IIIB open fractures of the extremities.

## Materials and methods

Study design and duration

This observational analytical study was conducted among patients who underwent wound closure by VAC in the treatment of Gustilo-Anderson Type IIIA/IIIB fractures of the limbs from December 2019 to July 2021.

Study area

This study was conducted at the Department of Orthopaedics, R. L. Jalappa Hospital, which is attached to Sri Devaraj Urs Academy of Higher Education and Research, Tamaka, Kolar.

Study population

All patients admitted to R. L. Jalappa Hospital and diagnosed with Gustilo-Anderson Type IIIA/IIIB fractures of the limbs who presented to the Department of Orthopaedics between December 2019 and July 2021 were considered for inclusion in the study.

Sample size calculation

The sample size was calculated based on the infection rate observed in a previous study [20], which reported an infection rate of 5.4% with an 8% absolute error. Given this prevalence rate, we calculated the minimum sample size needed for this study using the following formula: 3.84 × p × q/d^2^, where p is prevalence, q is the complement of p, and d is precision (i.e., 8% absolute error). The estimated sample size was 31. Expecting a drop rate of 10% during the follow-up, the final sample size was calculated as 34.

Sampling method

All consecutive patients admitted to R. L. Jalappa Hospital between December 2019 and July 2021 were included in the study.

Inclusion criteria

The inclusion criteria were patients aged between 18 and 60 years with Gustilo-Anderson Type IIIA/IIIB fractures of the limbs who presented to the hospital.

Exclusion criteria

Patients with a bone shortfall, bleeding diathesis, peripheral vascular disease, and anemia and those on immunosuppressive drugs, steroids, and anticoagulant therapy were excluded from the study.

Data collection

The status of the wound was graded using the Open Wound Scoring System [21], as shown in Table 1.

**Table 1:** Open wound scoring system.

Score (grade)	Status of wound
0	Closed wound
1	Skin or soft tissue defect
2	Bone, tendon, implant exposure (any one)
3	Bone, tendon, implant exposure (any combination of two or more)
4	Associated or residual infection

The Gustilo-Anderson classification system [9] was employed to evaluate fracture type among all participants, as shown in Table 2.

**Table 2:** Gustilo-Anderson classification system.

Gustilo type	Definition
I	Open fracture, clean wound, wound <1 cm in length
II	Open fractures, wounds longer than 1 cm without severe soft-tissue injury, flaps, and avulsions
III	An open segmental fracture or an open fracture with substantial soft-tissue laceration, injury, or loss. Open fractures caused by farm injuries, fractures requiring vascular repair, and fractures that have been open for more than 8 hours before treatment
IIIA	Despite severe soft-tissue laceration or destruction, Type III fractures have an adequate periosteal covering of the fractured bone
IIIB	Soft-tissue loss, periosteal stripping, and bone destruction characterize Type III fractures. Almost always necessitates a second soft-tissue covering operation (i.e., free or rotational flap)
IIIC	Regardless of the degree of soft-tissue injury, Type III fractures are accompanied by an artery injury that requires treatment

A total of 34 patients with open Gustilo-Anderson Type IIIA/IIIB limb fractures who satisfied the inclusion criteria and provided informed written consent were included in the study. A thorough clinical history, clinical examination, and investigations such as complete blood count, bleeding time, clotting time, blood grouping and Rh typing, culture sensitivity of the wound swabs, and X-rays were examined.

Internal fixation or external fixation of the fracture was completed within 72 hours or after a thorough debridement of compound fractures and achieving an unsoiled wound with skin and soft tissue loss, following which a sponge foam was applied to the wound. An adhesive drape was utilized to conceal the wound. Ultimately, the inner end of a suction tube was acquainted with the dead wound space with the outer end linked to the device. Wound dressings were replaced every 48 hours and negative pressure was applied non-stop for 10-14 days. The pressure was sustained at 125 mmHg continuously or intermittently for five minutes followed by two minutes off.

Patients were followed up for one month. They were advised to come to the hospital for scheduled check-ups after discharge. All participants were followed up during the study. Wounds were inspected weekly and the following measurements were documented: the presence of granulation tissue, healthy wound bed, reduction in wound drainage, and reduction in wound dimensions. Intervention therapy was completed when an adequate granulation base was accomplished.

The size of the wound was quantified by placing two pieces of transparent plastic sheets directly on the wound and marking the outline of the wound with a permanent ink marker on the outer sheet.

**Figure 1:**
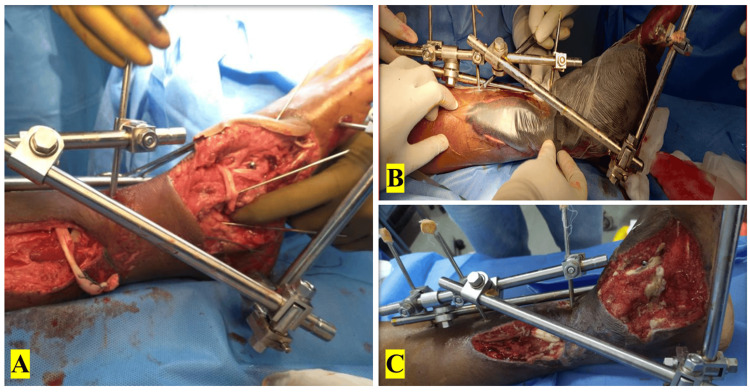
A case of open Type IIIB left distal third both bone fracture of the left leg with an open fracture of the left medial cuneiform and VAC dressing. (A) Intraoperative image after intervention by external fixation with percutaneous pinning. (B) VAC dressing application. (C) Post-VAC dressing removal. VAC: vacuum-assisted closure

The inner plastic sheet was cast off. The outer plastic sheet with the wound outlined was placed on calibrated graph paper. The size of the wound was then quantified by the greatest diameters, and the horizontal and vertical measurements were taken. VAC dressing was applied. Figure 1 shows the image of a case of open Type IIIB left distal third both bone fracture of the left leg with an open fracture of the left medial cuneiform.

Follow-up was done once a week until complete wound healing which could be either primary or by utilizing secondary interventions by tissue transfer. Antibiotics were given according to the culture and sensitivity report.

Ethical considerations

Ethics approval was obtained from the ethics committee at Sri Devaraj Urs Medical College & Research Institute (approval number: SDUMC/KLR/IEC/157/2019-20). All ethical guidelines were followed in the study. The data were only utilized for the anticipated purpose of the study. The dignity and welfare of participants were protected at all times from an ethical viewpoint. The study data remained confidential throughout the study.

Statistical analysis

Data were entered in an MS Excel spreadsheet (Microsoft Corp., Redmond, WA, USA) and analyzed using SPSS version 23 software (IBM Corp., Armonk, NY, USA). Discrete data such as gender, infection rate, and type of fractures were presented as frequency and percentages. Quantitative data such as age, wound size, duration of hospital stay, and reduction in flap procedures were presented as mean, standard deviation, and confidence interval. The McNemar test was used as a test of significance to compare the differences in proportion. A paired t-test was used to link the difference in wound size. A p-value less than 0.05 was considered statistically significant.

## Results

The mean age of the study participants was 37.06 years with an SD of 10.340, as shown in Table 3. Nearly 76.5% of the study population were males and the remaining were females, as shown in Table 4.

**Table 3:** Age distribution of the study participants (n = 34).

Age (year)	
Mean	37.06
Median	36.50
Mode	36
SD	10.340
Minimum	20
Maximum	59
Interquartile range	29.75–45.00

**Table 4:** Gender distribution of the study participants (n = 34).

Gender	Frequency	%
Female	8	23.5
Male	26	76.5
Total	34	100.0

Overall, 79.4% of trauma cases were due to road traffic accidents, and the remaining were due to workplace injuries, as shown in Table 5.

**Table 5:** Distribution of study participants according to the place of trauma (n = 34).

Type of injury	Frequency	%
Road traffic accident	27	79.4
Workplace injury	7	20.6
Total	34	100.0

Furthermore, 64.71% of the patients were classified as Gustilo-Anderson Type IIIB, and about 35.29% were classified as IIIA, as shown in Table 6. In the study participants, no neurovascular defects were noted. About 52.9% of the study participants were classified as Grade 2, and 35.3% were classified as Grade 3 by the Open Wound Grading System, as shown in Table 7.

**Table 6:** Distribution of study participants according to the type of fracture (n = 34).

Type of fracture	Frequency	%
IIIA	12	35.29
IIIB	22	64.71
Total	34	100.0

**Table 7:** Distribution of study participants according to the Open Wound Grading System (n = 34).

Open wound grading	Frequency	%
Grade 2	18	52.9
Grade 3	12	35.3
Grade 4	4	11.8
Total	34	100.0

Concerning the duration between the occurrence of trauma and initial debridement, the mean time difference was 13.76 minutes with an SD of 11.492 minutes, as shown in Table 8.

**Table 8:** Time between trauma and initial debridement.

The time between trauma and initial debridement	
Mean	13.76
Median	8.00
Mode	8
SD	11.492
Minimum	8
Maximum	48
Interquartile range	8.00–12.00

Regarding the difference in the surface area of wound dimension before and after the intervention, the mean surface area was 66.059 cm^2^ before the intervention and 27.97 cm^2^ after the intervention, as shown in Table 9.

**Table 9:** Area of wound dimension before and after intervention.

	Wound dimension before intervention	Wound dimension after intervention
Mean	66.059	27.97
Median	62.000	24.00
Mode	48.0	40
SD	28.8926	15.822
Minimum	32.0	8
Maximum	160.0	60
Interquartile range	46.000–80.000	16.00–40.00

Overall, 38.2% of the study participants underwent external fixation, and 32.4% of the individuals underwent percutaneous pinning after surgery, as shown in Table 10.

**Table 10:** Distribution of study participants according to the initial intervention after surgery (n = 34).

Initial intervention	Frequency	%
External fixation	13	38.2
External fixation with percutaneous pinning	1	2.9
Internal fixation	9	26.5
Percutaneous pinning	11	32.4
Total	34	100.0

Overall, 73.5% were managed by external fixation, and the remaining were managed by internal fixation, as shown in Table 11.

**Table 11:** Distribution of study participants according to the management by fixation (n = 34).

Management by fixation	Frequency	%
External fixation	25	73.5
Internal fixation	9	26.5
Total	34	100.0

Table 12 shows the mean duration of hospital stay, mean duration of VAC, and follow-up duration after intervention. Their mean duration was 19.38, 11, and 3.12 days, respectively.

**Table 12:** Outcome following the intervention among the study participants (n = 34). VAC: vacuum-assisted closure

Outcome following the intervention	Duration of hospital stay	Duration of VAC	Duration of follow-up
Mean	19.38	11.00	3.12
Median	20.00	12.00	3.00
Mode	12	6	3
SD	5.914	4.573	0.880
Minimum	10	6	2
Maximum	30	18	5
Interquartile range	14.00–24.00	6.00–12.50	2.00–4.00

The mean duration of wound healing duration was 18.47 days with an SD of 5.534 days, as shown in Table 13.

**Table 13:** Distribution of wound healing time among the study participants (n = 34).

Wound healing time	
Mean	18.47
Median	18.00
Mode	12
SD	5.534
Minimum	10
Maximum	28
Interquartile range	13.50–24.00

The most common comorbidity was diabetes mellitus (11.8%), and nearly 73.5% of the participants did not have any comorbidities (Table 14).

**Table 14:** Distribution of study participants according to their comorbidities (n = 34).

Comorbidities	Frequency	%
No comorbidity	25	73.5
Anemia	2	5.9
Diabetes	4	11.8
Hypertension	2	5.9
Thyroid disorder	1	2.9
Total	34	100.0

Skin maceration was seen among 21 patients, which was the only complication of VAC dressing in this study (Table 15).

**Table 15:** Distribution of study participants according to the wound complication (n = 34).

Wound complications	Frequency	%
None	13	38.2
Skin maceration	21	61.8
Total	34	100.0

In this study, the prevalence of deep infection among the study participants was 17.6%, as shown in Table 16.

**Table 16:** Deep infection among the study participants (n = 34).

Deep infection	Frequency	%
Yes	6	17.6
No	28	82.4
Total	34	100

Among the study participants, nearly 64.7% needed skin grafts for wound healing, as shown in Table 17.

**Table 17:** Need for skin grafts among the study participants (n = 34).

Skin graft	Frequency	%
Yes	22	64.7
No	12	35.3
Total	34	100

The prevalence of pre-VAC infection in this study was 91.2% (Table 18).

**Table 18:** Pre-VAC infection among the study participants (n = 34). VAC: vacuum-assisted closure

Pre-VAC infections	Frequency	%
Yes	31	91.2
No	3	8.8
Total	34	100

Regarding pre-VAC infection status, about 8.8% had no growth, and 11.8% had *Proteus *infection. The organisms responsible for pre-VAC infections are shown in Table 19.

**Table 19:** Organisms responsible for pre-VAC infections among the study participants (n = 34). VAC: vacuum-assisted closure

Pre-VAC infections	Frequency	%
No growth	3	8.8
*Acinetobacter*	3	8.8
*Escherichia coli*	2	5.9
*Escherichia coli*,* Acinetobacter*	1	2.9
*Klebsiella*	2	5.9
*Klebsiella*,* Escherichia coli*	1	2.9
*Klebsiella*,* Pseudomonas*	2	5.9
*Proteus*	4	11.8
*Pseudomonas*	2	5.9
*Staphylococcus aureus*	11	32.4
*Staphylococcus aureus*,* Pseudomonas*	2	5.9
*Staphylococcus aureus*,* Acinetobacter*	1	2.9
Total	34	100.0

The prevalence of post-VAC infections in the study was 17.6%, as shown in Table 20.

**Table 20:** Post-VAC infection among the study participants (n = 34). VAC: vacuum-assisted closure

Post-VAC infections	Frequency	%
Yes	6	17.6
No	28	82.4
Total	34	100

Regarding post-VAC infection status, about 82.4% had no growth and 5.9% had *Pseudomonas *and *Staphylococcus aureus* infection each. The organisms responsible for post-VAC infections are shown in Table 21.

**Table 21:** Disease organism responsible for post-VAC infections among the study participants (n = 34). VAC: vacuum-assisted closure

Post-VAC infection	Frequency	%
Pseudomonas	2	5.9
Staphylococcus aureus	2	5.9
Acinetobacter	1	2.9
Proteus	1	2.9
Nil	28	82.4
Total	34	100

The mean dimension of the wound before VAC therapy was 66.059 cm^2^ and the mean dimension of the wound after VAC therapy was 27.97 cm^2^. The difference in mean before and after the intervention was statistically significant according to the paired t-test (p < 0.001). Hence, VAC dressing helped decrease the wound size which was proved statistically. Table 22 shows the association of wound dimensions before and after the intervention by paired t-test.

**Table 22:** Association of wound dimension before and after intervention by paired t-test (n = 34).

Wound dimension	Mean	SD	Mean difference	P-value
Wound dimension before intervention	66.059	28.8926	33	<0.001
Wound dimension after intervention	27.97	15.822		

The prevalence of infection before VAC dressing was 80.6%, and the prevalence of infection after VAC dressing was 19.4%. The difference in proportion before and after the intervention was statistically significant (p < 0.001) according to the McNemar Test. Hence, VAC dressing helped decrease the rate of infection which was proven statistically. Table 23 shows the association between infections seen before and after the VAC intervention among the study participants by McNemar test.

**Table 23:** Association between infections before and after the VAC intervention among study participants by McNemar test (n = 34). VAC: vacuum-assisted closure

Infections before and after VAC dressing		Post-VAC infection, n (%)		P-value
		No	Yes	
Pre-VAC infection	No	3 (100)	0 (0)	<0.001
	Yes	25 (80.6)	6 (19.4)	

## Discussion

In this study, the prevalence of infection before VAC dressing was 80.6% and after VAC dressing was 19.4%. VAC helps in the speedy closure of the wound. The mean wound dimension before VAC therapy was 66.05 cm^2^ and after VAC therapy was 27.97 cm^2^. Hence, VAC dressing helps decrease the rate of infection.

The study participants were 37.06 ± 10.340 years old on average. This finding can be compared to the mean age of 38 years in a study by Joethy et al. from Singapore in 2013 [22]. In this study, males accounted for around 76.5% of the participants, while females accounted for the rest.

Road traffic accidents caused 79.4% of the trauma in this study, with the rest coming from employment injuries. This finding is similar to a prospective, randomized, interventional study conducted in India by Sinha et al. in 2013 [23], where the most common cause was road traffic accidents among 22 (73.33%) patients, followed by machinery injury among five (16.66%) patients, and an accidental fall from a height among three (10%) patients. Traumatic injuries are frequently associated with severe skin loss, which exposes tendons, bone, or metal, as well as wound management challenges. In many aspects, these injuries resemble chronic ulcerative lesions of the foot associated with ischemic illnesses such as diabetes mellitus. The rapid development of granulation tissue over the wound and blood vessels in and around the wound is necessary for wound healing. Furthermore, collagenase and metalloproteinase in interstitial fluid from open wounds limit local blood flow and disrupt wound healing. In this regard, NPWT is extremely successful at removing interstitial fluid.

Overall, 64.71% of the patients had Gustilo-Anderson Type IIIB fractures and 35.29% had Type IIIA fractures. External fixation accounted for over 73.5% of the cases, while internal fixation accounted for the rest. According to the open wound grading method, 52.9% of the study participants were classified as Grade 2 and 35.3% as Grade 3.

In this study, roughly 64.7% of the participants require skin grafts for wound healing. The requirement for free flap surgery was shown to be reduced by 30% in a comparison study of traditional dressings and NPWT for lawnmower injuries of the lower leg [24]. A significant reduction in the need for secondary soft tissue surgery is thought to be a significant benefit of NPWT [25]. Dedmond et al. further reported that Grade 3 wounds with an open tibial fracture healed without the requirement for a secondary soft tissue procedure such as a free flap [26].

Deep infection was noted in 17.6% of the patients according to a retrospective cohort study conducted by Blum et al. in 2012 [19]. When used for the dressing of traumatic wounds in open tibial fractures, NPWT reduces the chance of deep infection (8.4%). When the multivariate analysis was used to compensate for the Gustilo type, using NPWT reduced the probability of deep infection by about 80%. When utilized for the dressing of Gustilo Type IIIA/B fractures, a study conducted by Gill et al. in 2016 [1] showed that VAC reduced the chance of deep infection by 7%. As a result, VAC dressing is thought to minimize the chance of deep infection in Gustilo-type fractures.

Diabetes mellitus was the most commonly associated comorbidity in our study (11.8%), while roughly 73.5% of the patients had no comorbidities. This finding contrasts with a 2013 study by Joethy et al. in Singapore, which found that the prevalence of pre-existing comorbidities was 11-12% [22].

The average duration of VAC and follow-up was 11 days and 3.12 days, respectively. This finding was similar to that of Lee et al. in Korea [21], who found that the average duration of VAC therapy was 18.4 days (range: 11-29 days). Suman et al. [3]. from India reported an average period of VAC application of 10 days (range: 3-16). In the study, the mean duration of VAC dressing in patients with and without infection was 7.78 ± 0.42 days and 8.79 ± 1.19 days, respectively.

In this study, the mean wound healing time was 18.47 days, with an SD of 5.534 days. The duration of wound healing in VAC was found to be shorter with Type III tibial fractures, according to Hou et al. [27].

The mean delay between the occurrence of trauma and the beginning of debridement was 13.76 minutes, with an SD of 11.492 minutes. In contrast, Suman et al. [3] reported that the mean period between the trauma and the first debridement was 8.20 hours (range: 2-23).

The six-hour window between debridement and operative debridement of open lower-limb fractures was once thought to be critical in reducing infection rates, with this period dubbed the *golden period* for wound treatment. Regardless of the origins of the six-hour rule, debridement of open fractures within six hours of injury is a widely acknowledged standard of treatment, even though some studies have claimed that debridement during the golden period has no benefit [22].

Before VAC therapy, the average wound dimension was 66.059 cm^2^ and after VAC therapy, the average wound dimension was 27.97 cm^2^. According to the paired t-test, the difference in mean before and after the intervention was statistically significant (p = 0.001). As a result, VAC dressing was statistically proven to help reduce wound size. Hence, VAC is a good way to speed up wound closure.

Sinha et al. employed VAC for musculoskeletal injuries in a prospective, randomized, interventional trial from India in 2013 [23]. They discovered that following VAC, soft-tissue deficiencies shrank by more than 5 mm to 25 mm (a decrease of 26.66%); however, wound size shrank by less than 5 mm with normal wound care. They concluded that VAC care aided the quick production of granulation tissue, reducing healing time and secondary soft-tissue defect-covering procedures. Similar investigations by Joseph et al. [28], Morykwas and Argenta [14], and Morykwas et al. [15]. found that VAC was more successful than traditional wound dressings in lowering wound widths over time.

Infection was prevalent before VAC dressing at 80.6%, while it was prevalent after VAC dressing at 19.4%. According to the McNemar test, the difference in proportion before and after the intervention was statistically significant (p = 0.001). As a result, VAC dressing helped reduce the rate of infection, which was scientifically proven.

Sinha et al. employed VAC for open musculoskeletal injuries in a prospective, randomized, interventional trial in India [23] and found that after four days, 20% of cases showed no bacterial development, while on the eighth day, 60% of cases showed no bacterial growth. Similar investigations by Morykwas and Argenta [14], Banwell et al. [29], and Morykwas et al [15] showed that VAC would eliminate bacteria from infected wounds.

VAC therapy is a procedure that combines the advantages of both open and closed treatment while adhering to DeBakey’s principles of being brief, safe, and uncomplicated. It has been proven to be effective and therapeutic in wound healing. Although VAC therapy is not appropriate for all wounds, it can lead to considerable improvement in many instances. In severe cavities or extremely exudative wounds, VAC is most helpful. VAC is a useful tool for getting a wound to the point where it may be treated with more standard dressings or surgical reconstructive treatments.

Limitations

This study employed an observational study design. However, employing an experimental study design would have yielded more conclusive results. The ability of VAC dressing to control or decrease the bacterial load at the site of infection cannot be adequately explained within the confines of this observational study design. Unfortunately, this study lacked a control or comparison group, which would have allowed for a more comprehensive analysis of the effects of various types of dressing. The study was conducted using a sample size that was relatively smaller in comparison. The grading of wounds was simpler in this study. It is possible that smoking, which was not included in the study, could be a confounding factor that influences the severity of the injury. Despite this, the traumatic force plays a significant role in intensifying the severity of wounds. Because of the requirement of applying VAC, the cases had to undergo a prolonged period of hospitalization.

## Conclusions

The prevalence of infection before and after VAC dressing was 80.6% and 19.4%, respectively. VAC helped in speedy wound closure. The mean dimension of the wound before VAC therapy was 66.05 cm^2^ and after VAC therapy was 27.97 cm^2^. Hence, VAC dressing decreased the rate of infection. There is a cumulative body of data encouraging VAC as an adjunctive mode at all phases of treatment for Grade IIIA/B open fractures. There is a relationship between decreased infection rates and quick wound healing with VAC treatment.

## References

[1] Gill SP, Raj M, Kumar S, Singh P, Kumar D, Singh J, Deep A (2016). Early conversion of external fixation to interlocked nailing in open fractures of both bone leg assisted with vacuum closure (VAC) - final outcome. J Clin Diagn Res.

[2] Iheozor-Ejiofor Z, Newton K, Dumville JC, Costa ML, Norman G, Bruce J (2018). Negative pressure wound therapy for open traumatic wounds. Cochrane Database Syst Rev.

[3] Suman H, Krishna J, Sharma Y (2021). Role of VAC dressing in the large open fracture: a single center prospective study from Indore. Int J Res Rev.

[4] Court-Brown CM (2004). Reamed intramedullary tibial nailing: an overview and analysis of 1106 cases. J Orthop Trauma.

[5] Hoogendoorn JM, van der Werken C (2001). Grade III open tibial fractures: functional outcome and quality of life in amputees versus patients with successful reconstruction. Injury.

[6] Harley BJ, Beaupre LA, Jones CA, Dulai SK, Weber DW (2002). The effect of time to definitive treatment on the rate of nonunion and infection in open fractures. J Orthop Trauma.

[7] Khatod M, Botte MJ, Hoyt DB, Meyer RS, Smith JM, Akeson WH (2003). Outcomes in open tibia fractures: relationship between delay in treatment and infection. J Trauma.

[8] Jacob E, Erpelding JM, Murphy KP (1992). A retrospective analysis of open fractures sustained by U.S. military personnel during Operation Just Cause. Mil Med.

[9] Gustilo RB, Anderson JT (1976). Prevention of infection in the treatment of one thousand and twenty-five open fractures of long bones: retrospective and prospective analyses. J Bone Joint Surg Am.

[10] Cross WW 3rd, Swiontkowski MF (2008). Treatment principles in the management of open fractures. Indian J Orthop.

[11] Angelis S, Apostolopoulos AP, Kosmas L, Balfousias T, Papanikolaou A (2019). The use of vacuum closure-assisted device in the management of compound lower limb fractures with massive soft tissue damage. Cureus.

[12] Putnis S, Khan WS, Wong JM (2014). Negative pressure wound therapy - a review of its uses in orthopaedic trauma. Open Orthop J.

[13] Huang C, Leavitt T, Bayer LR, Orgill DP (2014). Effect of negative pressure wound therapy on wound healing. Curr Probl Surg.

[14] Argenta LC, Morykwas MJ (1997). Vacuum-assisted closure: a new method for wound control and treatment: clinical experience. Ann Plast Surg.

[15] Morykwas MJ, Argenta LC, Shelton-Brown EI, McGuirt W (1997). Vacuum-assisted closure: a new method for wound control and treatment: animal studies and basic foundation. Ann Plast Surg.

[16] Armstrong DG, Lavery LA, Abu-Rumman P, Espensen EH, Vazquez JR, Nixon BP, Boulton AJ (2002). Outcomes of subatmospheric pressure dressing therapy on wounds of the diabetic foot. Ostomy Wound Manage.

[17] Orgill DP, Austen WG, Butler CE (2004). Guidelines for treatment of complex chest wounds with negative pressure wound therapy. Wounds.

[18] Orgill DP (2004). Utilizing negative pressure wound therapy on open chest/sternotomy wounds. Ostomy Wound Manage.

[19] Blum ML, Esser M, Richardson M, Paul E, Rosenfeldt FL (2012). Negative pressure wound therapy reduces deep infection rate in open tibial fractures. J Orthop Trauma.

[20] Schlatterer DR, Hirschfeld AG, Webb LX (2015). Negative pressure wound therapy in grade IIIB tibial fractures: fewer infections and fewer flap procedures?. Clin Orthop Relat Res.

[21] Lee HJ, Kim JW, Oh CW (2009). Negative pressure wound therapy for soft tissue injuries around the foot and ankle. J Orthop Surg Res.

[22] Joethy J, Sebastin SJ, Chong AK, Peng YP, Puhaindran ME (2013). Effect of negative-pressure wound therapy on open fractures of the lower limb. Singapore Med J.

[23] Sinha K, Chauhan VD, Maheshwari R, Chauhan N, Rajan M, Agrawal A (2013). Vacuum assisted closure therapy versus standard wound therapy for open musculoskeletal injuries. Adv Orthop.

[24] Shilt JS, Yoder JS, Manuck TA, Jacks L, Rushing J, Smith BP (2004). Role of vacuum-assisted closure in the treatment of pediatric lawnmower injuries. J Pediatr Orthop.

[25] Mooney JF 3rd, Argenta LC, Marks MW, Morykwas MJ, DeFranzo AJ (2000). Treatment of soft tissue defects in pediatric patients using the V.A.C. system. Clin Orthop Relat Res.

[26] Dedmond BT, Kortesis B, Punger K (2006). Subatmospheric pressure dressings in the temporary treatment of soft tissue injuries associated with type III open tibial shaft fractures in children. J Pediatr Orthop.

[27] Hou Z, Irgit K, Strohecker KA, Matzko ME, Wingert NC, DeSantis JG, Smith WR (2011). Delayed flap reconstruction with vacuum-assisted closure management of the open IIIB tibial fracture. J Trauma.

[28] Joseph E (2000). A prospective randomized trial of vacuum-assisted closure versus standard therapy of chronic nonhealing wounds. Wounds.

[29] Banwell P, Withey S, Holten I (1998). The use of negative pressure to promote healing. Br J Plast Surg.

